# Independent and synergistic associations of cumulative body roundness index and its variability with fragility fracture risk

**DOI:** 10.1186/s12944-026-02923-4

**Published:** 2026-03-10

**Authors:** Zhou Yang, Lu Guo, Nan Zhang, Wenchao Yao, Yimin Liu, Wenqi Xu, Xinhao Fan, Xiaoli Hou, Jingyao Wang, Shuohua Chen, Jingyuan Gao, Lei Xing, Shouling Wu, Faming Tian

**Affiliations:** 1 School of Public Health, North China University of Science and Technology, Tangshan, Hebei China; 2 Kailuan General Hospital, Tangshan, Hebei China; 3 The School of Clinical Medicine, North China University of Science and Technology, Tangshan, Hebei China; 4 First Affiliated Hospital of Chongqing Medical University, Chongqing, China; 5 Department of General Practice, Affiliated Hospital of North China University of Science and Technology, Tangshan, Hebei China; 6 School of Public Health, Hebei Key Laboratory of Medical Engineering and Integrated Utilization of Saline alkali Land, North China University of Science and Technology, Bohai Road 21, Caofeidian Distict, Tangshan, 063200 Hebei China; 7 Department of Cardiology, Kailuan General Hospital, North China University of Science and Technology, No.57 Xinhua East Street, Tangshan, 063200 China

**Keywords:** Body roundness index, Cumulative exposure, Variability, Fragility fractures

## Abstract

**Background:**

The body roundness index (BRI) is a more comprehensive anthropometric measure than the body mass index (BMI) because it reflects the distribution of visceral fat more accurately. This study evaluated the separate and joint associations of cumulative BRI (cumBRI) exposure and its variability with the risk of fragility fractures in the Chinese population.

**Methods:**

This study included 47,240 individuals from the Kailuan Study who underwent three consecutive health examinations between 2006 and 2010 (2006–2007, 2008–2009, and 2010–2011) and were followed up until December 31, 2022. Cumulative BRI exposure was calculated using the time-weighted average (TWA), and variability in BRI was assessed using indices of variability. Cox models were used to estimate the hazard ratios (HRs) and 95% confidence intervals (CIs). Subgroup analysis and interaction testing performed. The C-index, discriminatory improvement (IDI), and net reclassification index (NRI) were used to assess fracture risk prediction.

**Results:**

During a median follow-up of 12.03 years, 725 participants developed fragility fractures. After adjusting for confounders, the HR for fragility fracture was 1.52 (95% CI 1.15–1.99) in the highest TWA BRI quartile, 1.38 (95% CI 1.08–1.75) in those with a cumulative burden of > 0, and 1.55 (95% CI 1.20-2.00) for those with ≥ 4 years of exposure duration. Greater BRI variability was associated with an increased risk of fragility fractures (HR 1.29, 95% CI 1.05–1.60]). The risk of fragility fractures was higher in individuals with a high TWA BRI and high BRI variability than in those with a low TWA BRI and low BRI variability. Combined measures of cumulative BRI and variability were better able to predict the risk of fracture than traditional metrics.

**Conclusions:**

Cumulative exposure to a high cumBRI and greater BRI variability in the BRI were independently associated with a higher incidence of fragility fractures, and their coexistence could may further increase the risk further. In terms of predicting the fracture risk, the combined effects of cumulative cumBRI exposure and variability predicting fracture risk are slightly superior to the predictive ability of obesity-related indices.

**Supplementary Information:**

The online version contains supplementary material available at 10.1186/s12944-026-02923-4.

## Background

 Global population aging has resulted in an increasing annual incidence of fragility fractures, which have now become a significant public health problem [1–3]. Each year, approximately 9 million fragility fractures occurring worldwide [4]. It is estimated that by 2050, the incidence of fragility fractures in China will have increased to 5.99 million cases, with associated costs reaching approximately 25.43 billion USD [5]. This will impose a heavy financial burden on families, society, and the healthcare systems [6]. Therefore, proactive identification of risk factors and interventions are crucial for effective primary prevention of fragility fractures.

The causes of fragility fracture are multifactorial, and many risk factors have been identified, including advanced age, low body weight, hypertension, diabetes, metabolic syndrome, and unhealthy lifestyle habits [7, 8]. Body weight is a crucial factor influencing metabolism and affects bone health at all stages of life [9]. Most of the research to date has focused on the impact of low body weight on bone health, while overlooking the effects of obesity. Obesity is a chronic metabolic disorder characterized by excessive accumulation of fat and metabolic changes, typically measured the by body mass index (BMI) [10]. An increasing amount of research is suggesting that individuals with a high BMI are at greater risk of bone fragility and fracture [11, 12]. However, BMI is not an ideal indicator of body fat content and distribution [13]. Thomas et al. proposed that the Body Roundness Index (BRI), which combines waist circumference (WC) and height, can be used to predict body fat and the volume of visceral fat tissue. Compared with traditional indicators, such as BMI, waist circumference, and hip circumference, BRI provides a better reflection of the proportions of body fat and visceral fat proportions [14]. BRI has also been found to be closely associated with a number of diseases [15]. Moreover, research has shown that a higher BRI is associated with lower bone mineral density (BMD) and a potentially greater risk of developing osteoporosis [16].

At present, there is a lack of research on the relationship between the BRI and the risk of fragility fractures. Considering that a single measurement of the BRI can be influenced by factors such as time and environment, it is inadequate for reflecting longitudinal changes and cumulative load. However, the cumulative BRI (cumBRI) and/or the variability in BRI may better reflect the impact of BRI on the risk of fragility fractures over time. Therefore, the aim of this study was to determine the individual and combined associations of cumBRI and BRI variability with the risk of fragility fractures in a Chinese population.

## Methods

### Study design and population

The Kailuan Study is a prospective investigation of a community–based cohort in Tangshan, China that aims to identify risk factors associated with chronic non–communicable diseases. The methodology and study design have been outlined elsewhere [17, 18]. Briefly, between 2006 and 2007, a total of 101,510 retirees in Kailuan underwent a baseline health examinations, with follow-up examinations at 2-yearly intervals thereafter. A total of seven follow-up assessments have been completed to date. During follow-up, participants completed questionnaires designed to gather information on lifestyle, demographics, and disease and drug history, along with anthropometric measurements and biochemical indicators. The annual incidence of fragility fractures was confirmed by review of diagnostic records and the medical insurance system of the Kailuan Group every 2 years until December 31, 2022.

The long-term cumBRI and BRI variability were calculated from the data for 58,869 participants who took part in three consecutive health examinations between 2006 and 2010 (2006–2007, 2008–2009, and 2010–2011). After exclusion of 11,143 individuals with missing data on waist circumference and height at any of the physical examinations, and 486 with a history of fragility fractures at baseline, data for 47,240 participants were included in the analysis (Figure S1). The study protocol was approved by the ethics committee of Kailuan General Hospital. All participants provided written informed consent.

### Calculation of BRI, cumBRI, time-weighted average (TWA) BRI, CumBRI burden, duration of high BRI, and BRI variability

1. BRI was calculated as $364.2-365.5\times\sqrt{1-\left[\frac{WC/\left(2\pi\right)}{0.5\times{height}}\right]^{2}}$, according to the formula developed by Thomas et al. [14].
2. CumBRI was calculated as follows:

CumBRI = [(BRI_06_+BRI_08_)/2 × (time_1–2_)] + [(BRI_08_+BRI_10_)/2 × (time_2–3_)] [19] where BRI_06_, BRI_08_, and BRI_10_ represent the BRI at the first, second, and third examinations, respectively, and time_1−2_, and time_2–3_ indicate the time intervals between consecutive visits in years.

(3) TWA BRI was calculated as cumBRI/(time1–3), where time1–3 indicates the total measurement period [20].
(4) The CumBRI burden was calculated using the following equation:

CumBRI burden= [(BRI_06_+BRI_08_) / 2 − cutoff value] × (time_1–2_) + [(BRI_08_+BRI_10_)/ 2 − cutoff value] × (time_2–3_). There is no recognized cutoff value for BRI. There is no recognized cutoff value for BRI. This research determined the optimal cutoff point for BRI by survival analysis using the maxstat package in R, with the resulting value calculated as 5.03 (Figure S2) [21].

(5) High BRI was defined as a BRI value of ≥ 5.03. The duration of high BRI was determined as the number of visits with high BRI values across the three checks, classified as 0 years (never had high BRI), 2 years (had high BRI once), or ≥ 4 years (had high BRI twice or three times) [22].
(6) BRI variability was assessed using four indices:

BRI variability was defined as the coefficient of variability (CV) (calculated as the standard deviation [SD]/mean BRI × 100%) of the BRI between visits [23]. Other measures of BRI variability, such as the SD, average real variability (ARV), and variation independent of mean (VIM), were also calculated [24]. We further stratified TWA BRI and BRI variability into 4 categories according to quartiles. The lowest quartile was used as the reference category in the subsequent analyses.

### Data collection and definitions

Demographic information (e.g., sex, age, and education), lifestyle factors (e.g., physical activity, alcohol consumption, and smoking status), and clinical characteristics (e.g., medication and disease history) were gathered by questionnaires. Details have been published previously [25, 26]. The participants underwent physical examinations conducted by trained field workers to measure their anthropometric parameters, including height, weight, waist circumference, and blood pressure (BP). Standing height, body weight, and, waist circumference, were recorded with participants barefoot and dressed in light indoor clothing using standard procedures and using calibrated instruments. Weight was accurate to 0.1 kg, and height was accurate to 1 mm. BMI was calculated as body mass (kg)/ height (m)^2^ [27]. Blood samples were collected in the morning following an overnight fast of 8 to 12 h and transferred into vacuum tubes containing EDTA. Triglycerides (TG), low-density lipoprotein cholesterol (LDL-C), high-density lipoprotein cholesterol (HDL-C), serum uric acid (UA), and fasting blood glucose (FBG) were analyzed using an enzymatic-colorimetric method. All biochemical indicators were analyzed using a Hitachi 747 auto-analyzer at the Kailuan General Hospital central laboratory.

Smoking was classified as “never”, “previous smoker” or “current smoker”. Alcohol consumption was classified as “never”, “previous drinker” or “current drinker”. Physical exercise was classified as “never or sometimes” or as “often.” Salt intake was dichotomized into high (> 10 g/day) or not high (≤ 10 g/day). Educational attainment was grouped into below high school, high school, and above high school. Hypertension was defined as either a self-reported history of hypertension, use of antihypertensive medications, or a blood pressure measurement of ≥ 140/90 mmHg. Diabetes was classified based on a self-reported history of diabetes, the use of hypoglycemic medication, or a fasting blood glucose level of ≥ 7.0 mmol/L.

### Assessment of outcomes

The primary endpoint was the number of fragility fracture events during follow-up. Follow-up started on the date of the baseline health examination in 2010 and continued to December 31, 2022. Fragility fractures were diagnosed based on the codes from the 10th Revision of the International Classification of Diseases and defined as fractures resulting from falls originating from a height at standing level or lower as arising without trauma during routine day-to-day activities [28]. The endpoint events were ascertained annually. To ensure accurate diagnosis of fragility fractures, specialists reviewed essential patient information and imaging records within the inpatient medical records system, while also collecting details regarding the cause of fractures. During a median follow-up of 12.03 years, a total of 725 incident fragility fracture events occurred. The number of events was sufficient to support stable estimation in multivariable Cox regression models and to ensure adequate precision of the main association results.

### Statistical analysis

Baseline data are presented as the mean ± SD, counts (percentages), or medians (P25, P75), as appropriate. To identify pairwise differences between groups, *p*-values were adjusted using the False Discovery Rate (FDR) method, with statistical significance set at FDR-adjusted *p* < 0.05. Superscript letters (a, b, c, d) indicate statistically significant pairwise differences, with a significant difference between groups that did not share the same letter. Cumulative incidence was determined using the Kaplan-Meier method. Cox proportional hazards models were used to investigate the associations of TWA BRI and BRI variability with the risk of incident fragility fractures. Hazard ratios (HRs) and corresponding 95% confidence intervals (CIs) were calculated. For the analysis of cumBRI, we evaluated the associations of TWA BRI values from three health examinations, cumulative BRI burden, and different durations of high-level BRI exposure with the risk of fragility fractures. For BRI variability, it was assessed using four indices (i.e.,CV, VIM, ARV, and SD). To adjust for potential confounding factors, model 1 was adjusted for sex (male or female), age (< 60 years or ≥ 60 years), smoking status (never, previous smoker, current smoker), drinking status (never, previous drinker, current drinker), heavy salt intake (yes or no), higher education (yes or no), physical exercise (yes or no), triglycerides (continuous), high-density lipoprotein cholesterol (continuous), low-density lipoprotein cholesterol (continuous), serum uric acid (continuous), use of antidiabetic medication (yes or no), and use of antihypertensive medication (yes or no), use of lipid-lowering medication (yes or no). Model 2 was additionally adjusted further for BMI (continuous) to determine whether the observed associations were independent of BMI. Model 3 was adjusted further for BRI in 2006–2007. We also utilized restricted cubic splines with three knots (at the 10th, 50th, and 90th percentiles) to explore the associations between TWA BRI, BRI variability, and fragility fractures across the various groups.

In addition, We also assessed the joint associations of TWA BRI and BRI variability with the risk of fragility fractures. Participants were divided into four combined groups based on the median of TWA BRI and BRI variability (greater than the median and less than or equal to the median) as follows:: “low TWA BRI and low variability,” “low TWA BRI and high variability,” (the reference group), “high TWA BRI and low variability,” and “high TWA BRI and high variability.” The reference group was the “low TWA BRI and low variability” group. Then, to assess if an effect modification exists, the analysis was stratified for age and sex and their interactions were examined. The C-index, discriminatory improvement (IDI), and net reclassification index (NRI) were used to analyze the ability to predict the risk of fragility fractures based on other relevant obesity-related indicators (height, body weight, body mass index, and waist circumference) as well as the cumulative, variability, and combined measures of the BRI.

Finally, we performed several sensitivity analyses to test the robustness of the results. First, we calculated the cumulative values of BRI to assess the combined effects of cumBRI and BRI variability on the risk of fragility fractures. Second, in order to avoid interference caused by antidiabetic drugs, we conducted a sensitivity analysis by excluding those who used antidiabetic medication. Third, we adjusted further for baseline diabetes status (yes/no) to determine whether the presence of diabetes influenced the association between BRI and fracture risk. Fourth, to rule out the potential for reverse causality, we removed participants who developed. Finally, Fine-Gray competing risk models were used. The statistical analyses were performed using SAS 9.4 software (SAS Institute Inc., Cary, NC, USA) and R 4.2.1 software (R Development Core Team, Vienna, Austria). A two-sided *p*-value of < 0.05 was considered statistically significant.

## Results

### Baseline characteristics

The mean age of the 47,240 study participants was 49.3 ± 11.8 years, with 80.2% (*n* = 37,896) being male. The average TWA BRI was 3.86 ± 1.00, and the median BRI variability (measured by the CV) was 15.3 [interquartile range 9.33, 23.2]. Table 1 shows the baseline characteristics of the study population by the combination of TWA BRI and BRI variability. There were some significant differences in baseline demographic and clinical characteristics and baseline measurements differed significantly among the four groups. Compared with the group that had low TWA BRI and low variability, participants in the other groups were more likely to have higher BMI, triglyceride, low-density lipoprotein cholesterol, systolic blood pressure, and fasting blood glucose levels, a higher prevalence of diabetes and hypertension, and to be on antihypertensive agents, lipid–lowering agents, and antidiabetic medications (including insulin, metformin, glinides, proprietary Chinese hypoglycemic medicines, and other hypoglycemic agents).The results of pairwise comparisons among the groups are shown in Table 1. Baseline characteristics grouped according to quartiles of TWA BRI and BRI variability are shown in Table S1 and Table S2, respectively, and are similar to the baseline characteristics in Table 1.

**Table 1:** Baseline characteristics of the study population according to combination of TWA BRI and BRI variability

Characteristics	Overall (*n* = 47,240)	Low TWA BRI, low variability (*n* = 11,083)	Low TWA BRI, high variability (*n* = 12,537)	High TWA BRI, low Variability (*n* = 12,537)	High TWA BRI, high variability (*n* = 11,083)	*P* value
Age, year	49.3 ± 11.8	46.6 ± 11.3^a^	47.4 ± 12.2^b^	51.0 ± 11.2^c^	52.1 ± 11.6^d^	< 0.001
Male, N (%)	37,896 (80.2)	9,020 (81.4)^a^	9,915 (79.1)^b^	10,352 (82.6)^c^	8,609 (77.7)^d^	< 0.001
Smoking status, N (%)						< 0.001
Never	28,870 (61.11)	6,373 (57.50)^a^	7,863 (62.72)^b^	7,332 (58.48)^c^	7,302 (65.88)^d^	-
Previous smoker	2,126 (4.50)	451 (4.07)	470 (3.75)	677 (5.40)	528 (4.76)	-
Current smoker	16,244 (34.39)	4,259 (38.43)	4,204 (33.53)	4,528 (36.12)	3,253 (29.35)	-
Drinking status, N (%)						< 0.001
Never	34,027 (72.03)	7,583 (68.42)^a^	9,343 (74.52)^b^	8,656 (69.04)^c^	8,445 (76.20)^d^	-
Previous drinker	6,386 (13.52)	1,684 (15.19)	1,629 (12.99)	1,813 (14.46)	1,260 (11.37)	-
Current drinker	6,827 (14.45)	1,816 (16.39)	1,565 (12.48)	2,068 (16.50)	1,378 (12.43)	-
≥Senior high school, N (%)	11,211 (23.7)	3,087 (27.9)^a^	3,499 (27.9)^a^	2,571 (20.5)^b^	2,054 (18.5)^c^	< 0.001
Intake salt, N (%)	5,088 (10.8)	1,239 (11.2)^a^	1,218 (9.72)^b^	1,510 (12.0)^c^	1,121 (10.1)^b^	< 0.001
Physical activity, N (%)	6,514 (13.8)	1,447 (13.1)^a, b^	1,642 (13.1)^b, c^	1,895 (15.1)	1,530 (13.8)^a, c^	< 0.001
BMI, kg/m^2^	25.2 ± 3.39	23.4 ± 2.34^a^	23.2 ± 2.75^b^	27.23 ± 2.86^c^	26.8 ± 3.29^d^	< 0.001
TG mmol/L M (P25, P75)	1.30 (0.93,1.92)	1.20 (0.87,1.69)^a^	1.10 (0.81, 1.53)^b^	1.55 (1.10, 2.34)^c^	1.40 (1.00, 2.10)^d^	< 0.001
HDL_C mmol/L	1.52 ± 0.42	1.58 ± 0.43^a^	1.57 ± 0.42^a^	1.47 ± 0.41^b^	1.45 ± 0.40^c^	< 0.001
LDL_C mmol/L	2.60 ± 0.80	2.58 ± 0.72^a^	2.54 ± 0.75^b^	2.65 ± 0.85^c^	2.64 ± 0.88^c^	< 0.001
UA µmmol/L	289.1 ± 86.5	277.2 ± 83.6^a^	271.7 ± 80.3^b^	308.1 ± 88.9^c^	299.1 ± 87.6^d^	< 0.001
FBG mmol/L	5.65 ± 1.48	5.44 ± 1.22^a^	5.43 ± 1.18^a^	5.92 ± 1.72^b^	5.83 ± 1.65^c^	< 0.001
SBP mmHg	131.4 ± 19.3	126.8 ± 17.7^a^	127.2 ± 18.9^a^	135.6 ± 18.9^b^	136.0 ± 19.8^b^	< 0.001
BRI_06_	3.77 ± 1.21	3.10 ± 0.54^a^	3.02 ± 0.93^b^	3.50 ± 0.79^c^	4.45 ± 1.47^d^	< 0.001
BRI (CV)	15.3 (9.33, 23.2)	9.54 (6.41, 12.4)^a^	23.6 (19.0, 30.6)^b^	9.15 (5.97, 12.4)^c^	22.9 (18.6, 29.6)^d^	< 0.001
BRI (ARV)	0.67 (0.40, 1.06)	0.34 (0.22, 0.47)^a^	0.87 (0.64, 1.17)^b^	0.48 (0.31, 0.68)^c^	1.27 (0.95, 1.73)^d^	< 0.001
BRI (SD)	0.56 (0.34, 0.87)	0.29 (0.19, 0.38)	0.72 (0.58, 0.95)	0.41 (0.27, 0.54)	1.03 (0.82, 1.35)	< 0.001
BRI (VIM)	0.59 (0.36, 0.93)	0.41 (0.27, 0.54)^a^	1.04 (0.83, 1.37)^b^	0.32 (0.21, 0.42)^c^	0.81 (0.65, 1.05)^d^	< 0.001
Time-weighted averaged BRI	3.86 ± 1.00	3.14 ± 0.46^a^	3.06 ± 0.50^b^	4.61 ± 0.72^c^	4.63 ± 0.86^c^	< 0.001
Cumulative BRI	5643.4 ± 1602.4	4552.7 ± 808.5^a^	4450.9 ± 855.6^b^	6765.5 ± 1268.8^c^	6813.7 ± 1431.5^d^	< 0.001
Diabetes, N (%)	5,150 (10.9)	705 (6.36)^a^	821 (6.55)^a^	2,034 (16.2)^b^	1,590 (14.4)^c^	< 0.001
Hypertension, N (%)	22,034 (46.6)	4,061 (36.6)^a^	4,454 (35.5)^a^	7,323 (58.4)^b^	6,196 (55.9)^c^	< 0.001
Antihypertensive agents, N (%)	6,952 (14.7)	1,037 (9.36)^a^	1,006 (8.02)^a^	2,858 (22.8)^b^	2,051 (18.5)^c^	< 0.001
Lipid-lowering agents, N (%)	497 (1.05)	75 (0.68)^a^	62 (0.49)^a^	215 (1.71)^b^	145 (1.31)^c^	< 0.001
Antidiabetic agents, N (%)	2,327 (4.93)	341 (3.08)^a^	376 (3.00)^b^	940 (7.50)^c^	670 (6.05)^d^	< 0.001
Insulin, N (%)	792 (1.68)	95 (0.86)^a^	110 (0.88)^a^	344 (2.74)^b^	243 (2.19)^c^	< 0.001
Metformin, N (%)	405 (0.86)	41 (0.37)^a^	45 (0.36)^a^	192 (1.53)^b^	127 (1.15)^c^	< 0.001
Glinides, N (%)	472 (1.00)	64 (0.58)^a^	58 (0.46)^a^	202 (1.61)^b^	148 (1.34)^b^	< 0.001
PCM hypoglycemic agents, N (%) n (%)	200 (0.42)	24 (0.22)^a^	23 (0.18)^a^	74 (0.59)^b^	79 (0.71)^b^	< 0.001
Others, N (%)	859 (36.91)	101 (29.62)a	117 (31.12)a	374 (39.79)b	267 (39.85)c	< 0.001

Data are presented as mean ± standardized deviation, median (p25, p75), or n(percentage)

Superscript letters (a, b, c, d) denote statistically significant pairwise differences between groups; groups not sharing the same letter differ significantly (False Discovery Rate– adjusted p < 0.05)

*SBP* systolic blood pressure, *BMI* body mass index, *FBG* fasting blood glucose, *LDL*-*C* low-density lipoprotein cholesterol, *HDL*-*C* high-density lipoprotein cholesterol, *TG* triglyceride, *UA* uric acid, *BRI* Body roundness index, *BRI*_*06*_ Body roundness index in 2006-2007, *ARV* average real variability, *VIM* variation independent of mean, *CV* coefficient of variation, *SD* standard deviation, *PCM* Proprietary Chinese medicine

### Association of TWA BRI, cumulative BRI, and duration of high BRI of fragility fracture

**Fig. 1:**
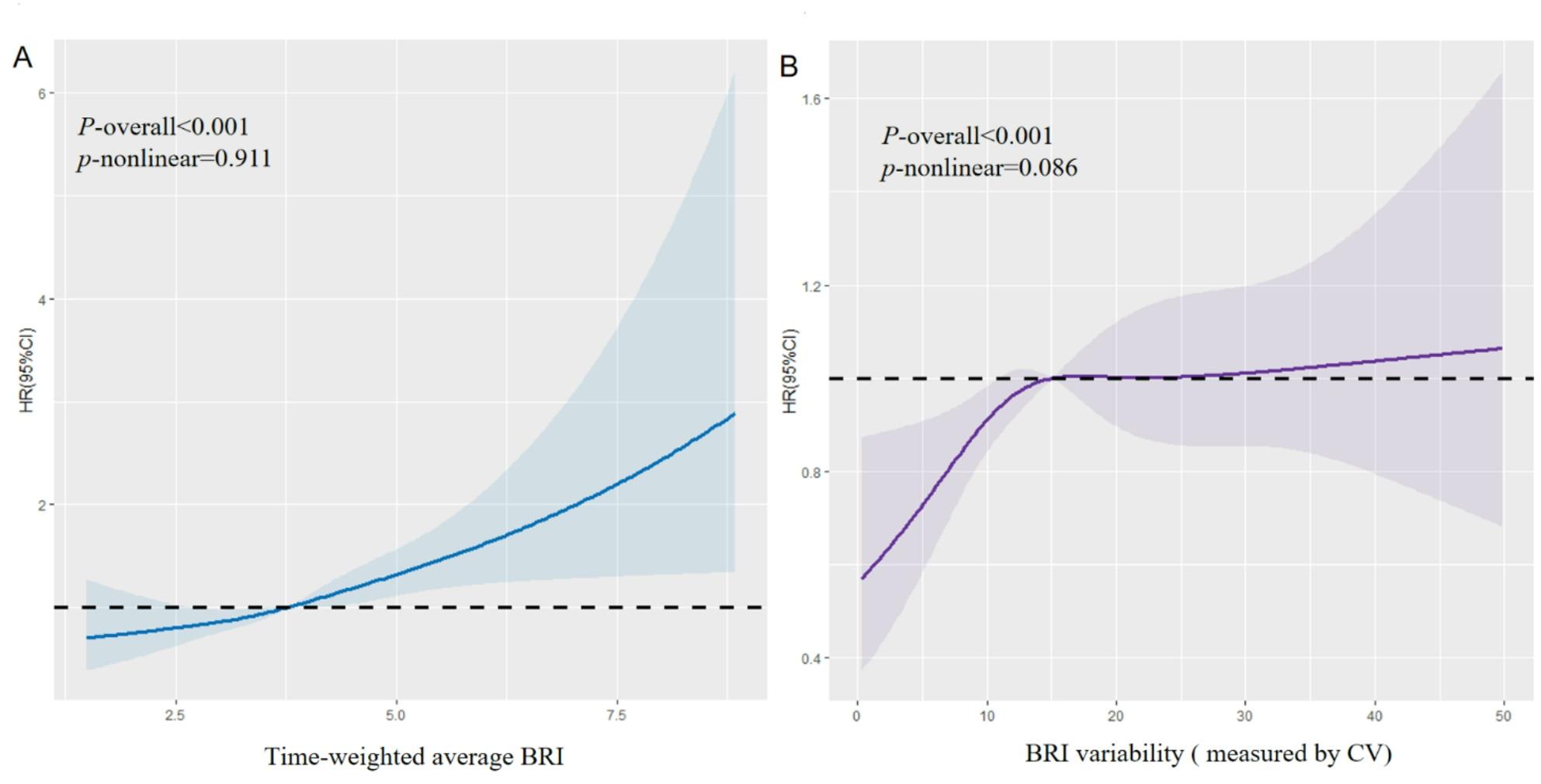
Dose–response relationship of TWA BRI and BRI variability with incidence of fragility fracture. **A**, Dose–response relationship between TWA BRI and fragility fracture. **B**, Dose–response relationship between BRI variability and fragility fracture.

During an average follow-up of 11.38 years, there were 725 cases of fragility fractures in total, of which 204 (28.14) were at hip fractures, 128 (17.66) were in an upper extremities, 163 (22.48) were lower extremities, 186 (25.66) were vertebral, rib, clavicular, scapular, or sternal fractures, and 44 (6.07%) were other fractures (Figure S3). The observed 12-year cumulative incidences of fragility fractures in the four groups were 1.35%, 1.50%, 1.48%, and 2.03%, respectively (Figure S4A). Table 2 shows the incidence density of fragility fractures and the multivariable HRs for the cumBRI indices. In model 1, after adjusting for potential covariates, the risk of fragility fracture was higher in participants in quartile 4 for TWA BRI (HR 1.38, 95% CI 1.10–1.72) than in those in quartile 1 for TWA BRI. The results remained essentially the same after correction for baseline (HR 1.52, 95% CI 1.15–1.99). In model 3, we further adjusted for the BRI level at enrollment to determine whether the association was affected by a single measurement of BRI and the association remained significant. Furthermore, the restricted cubic spline analysis revealed a linear relationship between TWA BRI and the risk of fragility fracture (Fig. 1A). Similar trends were seen for the cumulative burden of BRI and a high BRI duration. Compared with a cumulative burden of ≤ 0, the risk of fragility fracture increased 38% (HR 1.38, 95% CI 1.08–1.75) when the cumulative burden was > 0. In model 2, compared with participants in the group with no high BRI values (0 year), the HRs for risk of fragility fracture were 1.20 (95% CI 0.98–1.47) in the 2–year group, and 1.55 (95% CI2.00) in the ≥ 4 years group (Table 2).

**Table 2:** HRs and 95% CIs for the risk of fragility fracture stratified by cumulative BRI indexes

Index	Case/Total	Incidence rate^a^	Model 1	Model 2	Model 3
Time-weighted average BRI					
Quartile 1	152/11,810	1.11	Reference	Reference	Reference
Quartile 2	167/11,810	1.23	1.11 (0.89–1.39)	1.15 (0.92–1.45)	1.13 (0.89–1.43)
Quartile 3	187/11,810	1.40	1.25 (1.01–1.56)	1.33 (1.04–1.69)	1.29 (1.00-1.66)
Quartile 4	219/11,810	1.66	1.38 (1.10–1.72)	1.52 (1.15–1.99)	1.43 (1.06–1.96)
* P* for trend			0.003	0.002	0.014
Cumulative burden of BRI					
≤ 0	618/422,83	1.28	Reference	Reference	Reference
> 0	107/4,957	1.95	1.35 (1.09–1.67)	1.38 (1.08–1.75)	1.30 (1.01–1.68)
Time exposure duration					
0 year	475/34,069	1.22	Reference	Reference	Reference
2 years	141/8,256	1.53	1.17 (0.96–1.42)	1.20 (0.98–1.47)	1.18 (0.95–1.45)
≥ 4 years	109/4,915	2.01	1.45 (1.17–1.81)	1.55 (1.20-2.00)	1.49 (1.13–1.98)

Quartile 1: <3.168; Quartile 2: ≥3.168 ,<3.744; Quartile 3: ≥3.744 ,<4.387; Quartile 4: ≥4.387; BRI_06_: Body roundness index in 2006-2007; Incidence rate^a^ per 1000 person-years; Cut off value of BRI=5.03

Model 1: adjusted for age, sex, smoking status, drinking status, intake salt status, education, physical activity, TG, HDL_C, LDL_C, UA, Antidiabetic agents, Antihypertensive agents, Lipid-lowering agents

Model 2：further adjusted for BMI in baseline

Model 3：further adjusted for BRI_06_

### Association of BRI variability with of fragility fracture

The observed 12-year cumulative incidences of fragility fracture in the four groups (with BRI variability measured by CV) were 1.41%, 1.68%, 1.63%, and 1.95%, respectively (Figure S4 B) Table 3 shows the associations between each indicator of BRI variability and the risk of fragility fracture. After adjusting for potential covariates in model 2, participants in the highest quartile for BRI had a 29% higher risk of developing fragility fractures than those in the lowest quartile (HR 1.29, 95% CI 1.05–1.60). The associations persisted after further adjustment for BRI, with an HR of 1.29 (95% CI 1.05–1.59) for fragility fractures. Significant results were also observed for other BRI variability indices indices of BRI variability (i.e., VIM, ARV, and SD). Furthermore, a similar dose-response relationship was found for BRI variability and the risk of fragility fractures (Fig. 1B).

**Table 3:** HRs and 95% CIs for the risk of fragility fracture stratified by BRI variability

Index	Case/Total	Incidence rate^a^	Model 1	Model 2	Model 3
CV					
Quartile 1	156/11,810	1.15	Reference	Reference	Reference
Quartile 2	185/11,810	1.37	1.18 (0.96–1.46)	1.18 (0.95–1.46)	1.19 (0.96–1.47)
Quartile 3	178/11,810	1.33	1.13 (0.91–1.41)	1.13 (0.91–1.40)	1.14 (0.92–1.42)
Quartile 4	206/11,810	1.55	1.29 (1.04–1.59)	1.29 (1.05–1.60)	1.29 (1.05–1.59)
* P* for trend			0.037	0.032	0.034
VIM					
Quartile 1	160/11,810	1.18	Reference	Reference	Reference
Quartile 2	180/11,810	1.34	1.12 (0.90–1.39)	1.12 (0.91–1.39)	1.13 (0.92–1.40)
Quartile 3	199/11,810	1.48	1.24 (1.01–1.53)	1.25 (1.01–1.54)	1.27 (1.03–1.57)
Quartile 4	186/11,810	1.39	1.16 (0.93–1.43)	1.18 (0.95–1.47)	1.20 (0.96–1.49)
* P* for trend			0.119	0.083	0.061
ARV					
Quartile 1	149/11,810	1.09	Reference	Reference	Reference
Quartile 2	177/11,810	1.31	1.18 (0.95–1.47)	1.18 (0.95–1.47)	1.18 (0.95–1.47)
Quartile 3	190/11,810	1.42	1.25 (1.01–1.55)	1.25 (1.01–1.55)	1.24 (1.00-1.54)
Quartile 4	209/11,810	1.58	1.32 (1.07–1.63)	1.31 (1.06–1.62)	1.27 (1.02–1.58)
* P* for trend			0.011	0.013	0.029
SD					
Quartile 1	143/11,810	1.05	Reference	Reference	Reference
Quartile 2	182/11,810	1.34	1.26 (1.01–1.57)	1.26 (1.02–1.57)	1.25 (1.01–1.56)
Quartile 3	189/11,810	1.41	1.29 (1.04–1.61)	1.29 (1.04–1.61)	1.26 (1.01–1.57)
Quartile 4	211/11,810	1.60	1.39 (1.13–1.73)	1.39 (1.12–1.72)	1.32 (1.06–1.64)
* P* for trend			0.004	0.005	0.011

BRI Body roundness index; BRI_06_ Body roundness index in 2006–2007, Incidence rate^a^ per 1000 person-years, ARV average real variability, VIM variation independent of mean, CV coefficient of variation, SD standard deviation, CV, Quartile 1-Quartile 4: <9.330; ≥9.330 ,<15.268; ≥15.268 ,<23.243; ≥23.243; VIM, Quartile 1-Quartile 4: <0.357; ≥0.357 ,<0.594; ≥0.594 ,<0.926; ≥0.926; ARV, Quartile 1-Quartile 4: <0.395; ≥0.395 ,<0.668; ≥0.668 ,<1.064; ≥1.064; SD, Quartile 1-Quartile 4: <0.338; ≥0.338 ,<0.561; ≥0.561 ,<0.868; ≥0.868

Model 1: adjusted for age, sex, smoking status, drinking status, intake salt status, education, physical activity, TG, HDL_C, LDL_C, UA, Antidiabetic agents, Antihypertensive agents, Lipid-lowering agents

Model 2: further adjusted for BMI in baseline

Model 3: further adjusted for BRI_06_

### Joint associations of TWA BRI and BRI variability with risk of fragility fracture

**Fig. 2:**
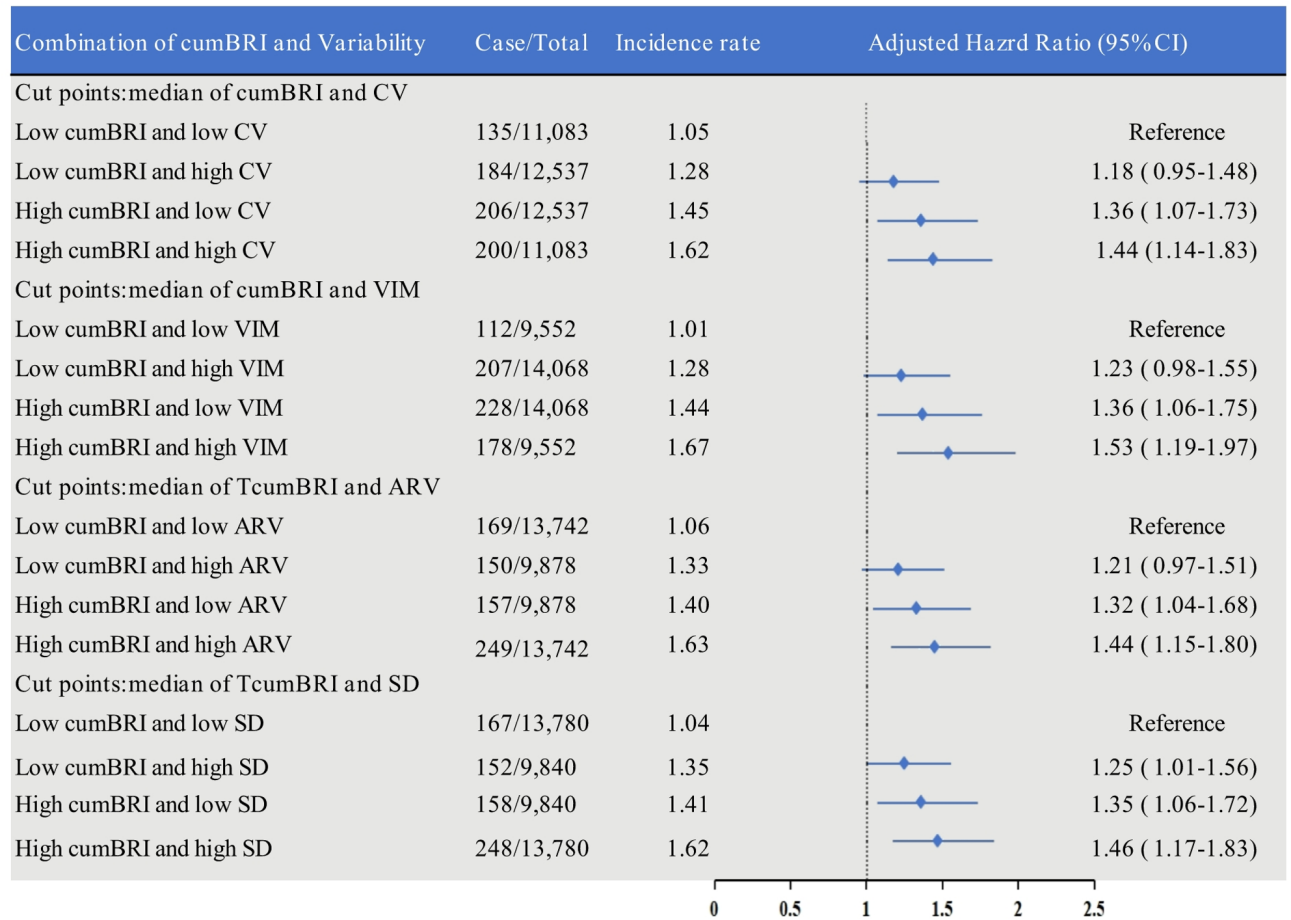
Stratified association between TWA BRI and BRI variability with fragility fracture Incidence. Note: median of TWA BRI, 3.770, median of variability CV, VIM, ARV, and SD are 15.268, 0.594, 0.668, and 0.561, Respectively. Model adjusted for age, sex, smoking status, drinking status, intake salt status, education, physical activity, TG, HDL_C, LDL_C, UA, antidiabetic agents, antihypertensive agents, lipid-lowering agents, and BMI in baseline.

The cumulative incidence rates for fragility fractures at 12 years were 1.28%, 1.56%, 1.78%, and 2.00%, respectively (Figure S4C). The combined effect of TWA BRI and BRI variability was considered, and the HRs for each group based on the median values for TWA BRI and BRI variability are shown in Fig. 2. After adjusting for confounders, participants with high TWA BRI and high CV values had the highest risk of fragility fracture (HR 1.44, 95% CI 1.14–1.82) when compared with those who had low TWA BRI and low CV. The results did not change after BRI variability was measured using VIM, ARV, and SD. When combined with variability measured by VIM, ARV, and SD, the risk of fracture in the “high TWA BRI and high VIM,” “high TWA BRI and high ARV.” and “high TWA BRI and high SD” groups was, respectively, 1.53, 1.44, and 1.46 times higher than in the corresponding groups with low values for these parameters.

### Stratified and prediction analyses

When stratified by age (< 60 years vs. ≥ 60 years) and sex (male vs. female), the fracture risk associated with BRI was higher in individuals aged < 60 and in men (Figures S5 and S7). However, the effect size of TWA BRI and/or its variability on fragility fractures had no significant interaction effect across the subgroups (*p* > 0.05 for all interactions) (Figures S5, S6, and S7). Addition of TWA BRI and BRI variability to the main model (model 2) improved the predictive value for risk of fragility fracture to a greater extent than addition of baseline BRI, height, weight, waist circumference, or BMI. The C–index increased by 0.7% from the 60.4% recorded for the main model. The discriminatory improvement was 0.0002 (0.0001–0.0003), indicating a 0.02% increase in the probability of correct prediction. The continuous net reclassification index was 0.117 (0.043–0.190), and the overall correct classification ability improved by 11.7% (Table S4).

### Sensitivity analyses

To assess the robustness of our results, we calculated the cumBRI to determine the combined effects of cumBRI and BRI variability on the risk of fragility fractures. As shown in Table S3, the results were consistent with the main findings. Furthermore, the associations of TWA BRI and BRI variability with the risk of fragility fractures were essentially unchanged after excluding participants who used antidiabetic medication, those who developed fragility fractures within the first year of follow-up, and after performing a Fine–Gray competing risks analysis (Tables S5, S6, and S7). Additional adjustment for baseline diabetes status did not materially alter the association between the BRI and the risk of fragility fracture (Tables S5, S6, and S7).

## Discussion

This large prospective population–based cohort study is the first to explore the separate and joint associations of cumBRI and its variability with the risk of fragility fractures. We found that an elevated cumBRI level and prolonged exposure to high BRI significantly increased the risk of fragility fractures independent of BMI and single measurements of BRI. Furthermore, higher BRI variability was associated with an increased risk of fragility fractures. We also observed a linear dose–response relationship between cumBRI, BRI variability, and the risk of fragility fractures. Finally, the ability of the cumBRI and BRI variability to predict the risk of fragility fractures when combined may be higher than that of other traditional obesity metrics.

The relationship between obesity and fractures remains controversial. Obesity has long been considered to have a protective effect on bone [29]. However, recent research indicates that the influence of adiposity on skeletal microarchitecture may depend on fat distribution [30, 31]. Accumulating evidence shows both general obesity (assessed by BMI) and abdominal obesity (measured by waist circumference) are closely associated with the risk of fragility fractures [32–34]. In recent years, it has been found that the BRI, a novel body index, has a notable inverse relationship with bone mineral density. Ding et al. showed that an increment in BRI of one unit was associated with a reduction in total bone mineral density of 0.0313 g/cm² (*p* < 0.0001) [16]. Nevertheless, to date, the relationship between BRI and the risk of fractures has been unclear. Some of the above reports support our findings. After correction for confounders, our results indicate significant associations of higher cumBRI levels and prolonged exposure to high BRI with an increased risk of fragility fractures independent of the BMI. Furthermore, we found that as cumBRI levels increased, the risk of fragility fractures also increased. This finding suggests that the BRI is not only an indicator of obesity but may also reveal the independent effects of type of fat distribution on bone health, with cumulative exposure providing more information on the risk of fragility fractures. Electronic health records allow for rapid integration of data across multiple time points, and their use can provide valuable evidence for the prevention of fragility fractures.

The present study is also the first to identify an association between BRI variability and the risk of fragility fractures. We found that high variability in BRI (i.e., significant fluctuations in body shape over time) was closely associated with an increased risk of fragility fractures. This risk was 1.29 times higher in the fourth quantile of BRI variability than in the first quantile (Table 3). This finding suggests that instability in body shape may be an important factor influencing bone health. Fluctuations in body shape may reflect changes in nutritional status, weight, or lifestyle, all of which can negatively impact bone density and strength, thereby increasing the risk of fragility fractures. This finding is consistent with previous reports. Meyer et al. noted that the relative risk of fractures was higher in participants with the highest weight variability (2.07, 95% CI 1.24–3.46 for women; 2.70, 95% CI 1.25–5.86 for men) after 11.6 years of follow-up [35]. Lee et al. similarly found that high variability in body weight was associated with a higher incidence of hip fractures in patients with diabetes [36].

Notably, given the combined effect of TWA BRI and its variability, our results indicate that the risk of fragility fractures was 44% higher in participants with high TWA BRI and high BRI variability than in the reference group (Fig. 2). This finding suggests that the combined effect of cumBRI and BRI variability may predict the risk of fragility fractures more accurately. Previous research has found that a stable BMI, rather than one that is fluctuating widely, and avoidance of central adiposity may help to decrease the risk of fracture [37, 38]. Our results also indicate that cumBRI and its variability are better predictors of the risk of fracture than individual factors such as height, weight, waist circumference, and BMI. This result is consistent with previous reports. Thomas et al. reported that prediction of body fat percentage and visceral fat volume was slightly better when using the BRI than when using traditional indicators, such as BMI, waist circumference, or hip circumference [14]. Tian et al. found that the BRI had the best predictive ability for identifying hypertension, diabetes, dyslipidemia, hyperuricemia, and metabolic syndrome [39, 40]. There is currently a lack of research on the role of the BRI in the prediction of fracture risk, and our data do not include direct measurements of related fat composition. Future studies should explore the relationship between BRI and fat composition to gain a better understanding of the potential of the BRI to predict fracture risk.

The results of this study have important clinical implications. First, assessment of cumBRI and its variability could provide a new tool for early screening of individuals at high risk for fragility fractures, especially when traditional BMI measurements fail to identify high–risk individuals. Second, for populations with prolonged exposure to a high BRI level and high BRI variability, early personalized intervention strategies, such as weight management, exercise, and nutritional support, may be necessary to reduce the incidence of fragility fractures.

This study found that both the cumulative BRI and its fluctuations over time were associated with an increased risk of fragility fractures, although the precise physiological mechanisms underpinning this relationship remain unclear. Our findings and previous reports suggest that several mechanisms may contribute to this association. First, obese individuals are at an increased risk of falls. Obesity may lead to biomechanical impairments, such as reduced lower limb muscle mass, increased foot load, and impaired postural control, which can decrease postural stability and increase susceptibility to falls [40]. Second, adiposity is associated with chronic low–grade inflammation. Higher C–reactive protein, tumor necrosis factor–alpha, and interleukin–6 levels are observed in individuals with central and visceral obesity, and this inflammatory response may be one of the factors contributing to the accelerated bone loss associated with obesity [41, 42]. Third, an increase in fatty bone marrow may impact bone health and contribute to development of the osteoporosis and increased bone fragility. Obesity increases the number of adipocytes in the bone marrow, alters their metabolic activity, and promotes differentiation of mesenchymal stem cells in bone marrow into adipocytes rather than osteoblasts [43, 44].

This study has some strengths. We used data from a large prospective cohort with complete longitudinal data. Height, weight, and waist circumference were measured by trained healthcare professionals, ensuring good reliability and accuracy. We also used data from multiple time points to analyze the cumulative and variability effects of BRI and their joint associations and performed sensitivity analyses to enhance the reliability of our findings.

However, this research also had some limitations. First, even after adjusting for known confounders, there may still have been factors that were unmeasured or incompletely controlled for (e.g., environmental factors, calcium supplementation, or use of estrogen or other medications) that could potentially have influenced the results. Second, the study population was derived from the Kailuan community, where there are more men than women, potentially limiting the generalizability of our findings. Third, given that the study population included a proportion of younger and middle–aged individuals at baseline, the incidence of fragility fractures was relatively low, which may have attenuated the magnitude of the observed associations to some extent; however, the direction of the associations is unlikely to have been affected. Fourth, this study was based on the Chinese Kailuan cohort, in which participants were predominantly adults with a relatively homogeneous ethnic background. Given that there may be biological differences in body fat distribution, skeletal structure, and susceptibility to fragility fractures across ethnic groups, the applicability of our findings to other populations warrants further investigation. Finally, this was an observational study, which means that causal relationships cannot be established. Therefore, controlled trials are needed to further explore the mechanistic relationship between BRI and fracture risk and help us to better understand the complex interactions between obesity phenotype and fracture.

## Conclusions

This study suggests that a higher cumBRI and greater variability in the BRI are significantly associated with an increased risk of fragility fractures, and their coexistence may further exacerbate this risk. Our findings highlight the importance of not only reducing long–term cumulative exposure to BRI but also minimizing significant fluctuations. As an anthropometric measurement, the BRI may be more sensitive than traditional BMI in terms of reflecting body fat distribution, particularly accumulation of abdominal fat, and could help in early identification of populations at high risk for fragility fractures. 

## Supplementary Information

Supplementary Material 1.

## Data Availability

The data that support the findings of this study are available on request from the corresponding author. The data are not publicly available due to privacy or ethical restrictions.

## Abbreviations

BRI: Body roundness index
BMI: Body mass index
cumBRI: Cumulative BRI
TWA: Time-weighted average
HR: Hazard ratio
CI: Confidence interval
IDI: Discriminatory improvement
NRI: Net reclassification index
WC: Waist circumference
BMD: Bone mineral density
CV: Coefficient of variability
SD: Standard deviation
ARV: Average real variability
VIM: Variation independent of mean
BP: Blood pressure
FBG: Fasting blood glucose
HDL-C: High-density lipoprotein cholesterol
LDL-C: Low-density lipoprotein cholesterol
CRP: C-reactive protein
TG: Triglyceride
TC: Total cholesterol
UA: Uric acid
TNF-α: Tumor necrosis factor-alpha
IL-6: Interleukin-6
