# Supplementary material for: Independent and synergistic associations of cumulative body roundness index and its variability with fragility fracture risk
Source: Lipids Health Dis. 2026 Mar 10;25:111. doi: 10.1186/s12944-026-02923-4 (PMC13085323; doi:10.1186/s12944-026-02923-4)
Supplement: Supplementary file 1 — Supplementary Material 1. [file 12944_2026_2923_MOESM1_ESM.docx]

**Independent and Synergistic Associations of the Cumulative Body Roundness Index and Its Variability with the risk of fragility fractures**

**Supplemental Materials**

**Supplement Table 1.** Baseline characteristics of the study population according to TWA BRI

**Supplement Table 2**. Baseline characteristics of the study population according to BRI variability (Measured by CV)

**Supplement Table 3**. Joint associations of cumBRI and BRI variability with the incidence of fragility fractures

**Supplement Table 4.** Reclassification and discrimination statistics

**Supplement Table 5.** Sensitivity analyses for the association between TWA BRI and the risk of fragility fractures

**Supplement Table 6.** Sensitivity analyses for the association between variability of BRI and the risk of fragility fractures

**Supplement Table 7.** Sensitivity analyses for joint associations between TWA BRI and BRI variability with fragility fracture incidence

**Supplement Figure 1.** Flow chart of study participants

**Supplement Figure 2**. Distribution of BRI and determination of the optimal cutpoint using maximally selected rank statistics.

**Supplement Figure 3.** Distribution of fractures location

**Supplement Figure 4.** Cumulative incidence of fragility fractures

**Supplement Figure 5.** Stratified association between cumulative BRI indexes with fragility fracture incidence by age and sex

**Supplement Figure 6.** Stratified association between BRI variability indexes with fragility fracture incidence by age and sex

**Supplement Figure 7.** Stratified analyses for joint associations between TWA BRI and BRI variability with fragility fracture incidence by age and sex

**Supplement Table1.** Baseline characteristics of the study population according to TWA BRI

| **Characteristics** | **Quantile 1**  **(n=11,810)** | **Quantile 2**  **(n=11,810)** | **Quantile 3**  **(n=11,810)** | **Quantile 4**  **(n=11,810)** | **P value** |
| --- | --- | --- | --- | --- | --- |
| Age, year | 45.2 ± 12.0^a^ | 48.8 ± 11.2^b^ | 50.3 ± 11.2^c^ | 52.8 ± 11.5^d^ | <0.001 |
| Male, N (%) | 9,133 (77.3)^a^ | 9,802 (83.0)^b^ | 9,790 (82.9)^b^ | 9,171 (77.7)^a^ | <0.001 |
| Smoking status, N (%) |  |  |  |  | <0.001 |
| Never | 7,178 (60.78)^a^ | 7,058 (59.76)^b^ | 7,136 (60.42)^c^ | 7,498 (63.49)^d^ | - |
| Previous smoker | 415 (3.51) | 506 (4.28) | 591 (5.00) | 614 (5.20) | - |
| Current smoker | 4,217 (35.71) | 4,246 (35.95) | 4,083 (34.57) | 3,698 (31.31) | - |
| Drinking status, N (%) |  |  |  |  | <0.001 |
| Never | 8,613 (72.93)^a^ | 8,313 (70.39)^b^ | 8,336 (70.58)^c^ | 8,765 (74.22)^d^ | - |
| Previous drinker | 1,648 (13.95) | 1,665 (14.10) | 1,616 (13.68) | 1,457 (12.34) | - |
| Current drinker | 1,549 (13.12) | 1,832 (15.51) | 1,858 (15.73) | 1,588 (13.45) | - |
| ≥Senior high school, N (%) | 3,814 (32.3)^a^ | 2,772 (23.5)^b^ | 2,553 (21.6)^c^ | 2,072 (17.5)^d^ | <0.001 |
| Intake salt, N (%) | 1,197 (10.1)^a^ | 1,260 (10.7)^b^ | 1,274 (10.8)^c^ | 1,357 (11.5)^a^ | <0.001 |
| BMI, kg/m^2^ | 22.3 ± 2.36^a^ | 24.4 ± 2.31^b^ | 25.9 ± 2.50^c^ | 28.2 ± 3.16^d^ | <0.001 |
| Physical activity, N (%) | 1,465 (12.4)^a^ | 1,624 (13.8)^b^ | 1,674 (14.2)^b,c^ | 1,751 (14.8)^c^ | <0.001 |
| TG mmol/L M (P25, P75) | 1.08 (0.78, 1.45)^a^ | 1.24 (0.90, 1.79)^b^ | 1.40 (1.00, 2.11)^c^ | 1.56 (1.10, 2.34)^d^ | <0.001 |
| HDL_C mmol/L | 1.62 ± 0.44^a^ | 1.53 ± 0.41^b^ | 1.48 ± 0.41^c^ | 1.44 ± 0.40^d^ | <0.001 |
| LDL_C mmol/L | 2.51 ± 0.69^a^ | 2.61 ± 0.78^b^ | 2.64 ± 0.83^c^ | 2.65 ± 0.90^c^ | <0.001 |
| UA μmmol/L | 265.9 ± 77.9^a^ | 282.7 ± 85.0^b^ | 298.2 ± 88.3^c^ | 309.5 ± 88.3^d^ | <0.001 |
| FBG mmol/L | 5.32 ± 1.05^a^ | 5.54 ± 1.32^b^ | 5.74 ± 1.60^c^ | 6.01 ± 1.76^d^ | <0.001 |
| SBP mmHg | 123.6 ± 17.4^a^ | 130.4 ± 18.7^b^ | 133.3 ± 18.8^c^ | 138.2 ± 19.6^d^ | <0.001 |
| BRI_06_ | 3.89 ± 0.95^a^ | 3.79 ± 1.01^b^ | 3.68 ± 1.09^c^ | 3.70 ± 1.66^d^ | <0.001 |
| BRI (CV) | 17.1 (10.6, 25.5)^a^ | 15.06 (9.30, 22.9)^b^ | 14.69 (8.83, 22.3)^c^ | 14.39 (8.76, 22.2)^b^ | <0.001 |
| BRI (ARV)  BRI (SD) | 0.54 (0.33, 0.84)^a^  0.46 (0.28, 0.69) | 0.62 (0.37, 0.96)^b^  0.52 (0.32, 0.79) | 0.71 (0.41, 1.12)^c^  0.59 (0.36, 0.90) | 0.85 (0.51, 1.37)^d^  0.72 (0.44, 1.11) | <0.001  <0.001 |
| BRI (VIM)  Time-averaged BRI  Cumulative BRI  Diabetes, N (%) | 0.81 (0.50, 0.21)^a^  2.71 ± 0.37  3922.4 ± 660.3  524 (4.44) | 0.61 (0.38, 0.93)^b^  3.48 ± 0.17  5074.9 ± 543.5  1,002 (8.48) | 0.55 (0.33, 0.83)^c^  4.07 ± 0.18  5963.6 ± 634.4  1410 (11.94) | 0.47 (0.28, 0.73)^d^  5.16 ± 0.78  7612.6 ± 1367.9  2214 (18.75) | <0.001  <0.001  <0.001  <0.001 |
| Hypertension, N (%) | 3,407 (28.9)^a^ | 5,108 (43.3)^b^ | 6,042 (51.2)^c^ | 7,477 (63.3)^d^ | <0.001 |
| Diabetes,N (%) | 524 (4.44)^a^ | 1,002 (8.48)^b^ | 1,410 (11.94)^c^ | 2,214 (18.75)^d^ | <0.001 |
| Antihypertensive agents, N (%) | 722 (6.11)^a^ | 1,321 (11.2)^b^ | 1,905 (16.1)^c^ | 3,004 (25.4)^d^ | <0.001 |
| Lipid-lowering agents, N (%) | 38 (0.32)^a^ | 99 (0.84)^b^ | 135 (1.14)^c^ | 225 (1.91)^d^ | <0.001 |
| Antidiabetic agents, N (%) | 282 (2.39)^a^ | 435 (3.68)^b^ | 606 (5.13)^c^ | 1,004 (8.50)^d^ | <0.001 |
| Insulin, N (%) | 54 (0.46)^a^ | 151 (1.28)^b^ | 204 (1.73)^c^ | 383 (3.24)^d^ | <0.001 |
| Metformin, N (%) | 20 (0.17)^a^ | 66 (0.56)^b^ | 107 (0.91)^c^ | 212 (1.80)^d^ | <0.001 |
| Glinides, N (%) | 34 (0.29)^a^ | 88 (0.75)^b^ | 117 (0.99)^c^ | 233 (1.97)^d^ | <0.001 |
| PCM hypoglycemic agents, N (%) | 14 (0.12)^a^ | 33 (0.28)^b^ | 48 (0.41)^b^ | 105 (0.89)^c^ | <0.001 |
| Others, N (%) | 57 (20.21)^a^ | 161 (37.01)^b^ | 222 (36.63)^c^ | 419 (41.73)^d^ | <0.001 |

Data are presented as mean ± standardized deviation, median (p25, p75), or n(percentage). SBP, systolic blood pressure; BMI, body mass index; FBG,

fasting blood glucose; LDL-C, low-density lipoprotein cholesterol; HDL-C, high-density lipoprotein cholesterol; TG, triglyceride; UA, uric acid; BRI, Body

roundness index; BRI_06_, Body roundness index in 2006-2007; ARV, average real variability; VIM, variation independent of mean; CV, coefficient of variation;

SD, standard deviation; PCM, Proprietary Chinese medicine; Superscript letters (a, b, c, d) denote statistically significant pairwise differences between groups;

groups not sharing the same letter differ significantly (False Discovery Rate–adjusted *p* < 0.05).

**Supplement Table2**. Baseline characteristics of the study population according to BRI variability (Measured by CV)

| **Characteristics** | **Quantile 1**  **(n=11,810)** | **Quantile 2**  **(n=11,810)** | **Quantile 3**  **(n=11,810)** | **Quantile 4**  **(n=11,810)** | **P value** |
| --- | --- | --- | --- | --- | --- |
| Age, year | 48.8 ± 11.2^a^ | 49.1 ± 11.5^a,b^ | 49.2 ± 12.0^b^ | 50.0 ± 12.4^c^ | <0.001 |
| Male, N (%) | 9,772 (82.7)^a^ | 9,600 (81.2)^b^ | 9,462 (80.3)^c^ | 9,062 (76.7)^d^ | <0.001 |
| Smoking status, N (%) |  |  |  |  | <0.001 |
| Never | 6,679 (56.55)^a^ | 7,026 (59.49)^b^ | 7,314 (61.93)^c^ | 7,851 (66.48)d | - |
| Previous smoker | 554 (4.69) | 574 (4.86) | 547 (4.63) | 451 (3.82) | - |
| Current smoker | 4,577 (38.76) | 4,210 (35.65) | 3,949 (33.44) | 3,508 (29.70) | - |
| Drinking status, N (%) |  |  |  |  | <0.001 |
| Never | 7,905 (66.93)^a^ | 8,334 (70.57)^b^ | 8,685 (73.54)^c^ | 9,103 (77.08)^d^ | - |
| Previous drinker | 1,858 (15.73) | 1,639 (13.88) | 1,564 (13.24) | 1,325 (11.22) | - |
| Current drinker | 2,047 (17.33) | 1,837 (15.55) | 1,561 (13.22) | 1,382 (11.70) |  |
| ≥Senior high school, N (%) | 2,836 (24.0)^a^ | 2,822 (23.9)^a^ | 2,843 (24.1)^a^ | 2,710 (23.0)^a^ | <0.001 |
| Intake salt, N (%) | 1,362 (11.5)^a^ | 1,387 (11.7)^a^ | 1,189 (10.1)^b^ | 1,150 (9.74)^b^ | <0.001 |
| BMI, kg/m^2^ | 25.5 ± 3.19^a^ | 25.4 ± 3.28^b^ | 25.1 ± 3.38^c^ | 24.6 ± 3.61^d^ | <0.001 |
| Physical activity, N(%) | 1,629 (13.8) | 17,13 (14.5)^a^ | 1,627 (13.8) | 1,545 (13.1)^b^ | <0.001 |
| TG mmol/L M (P25, P75) | 1.40 (1.00, 2.10)^a^ | 1.34 (0.96, 1.99)^b^ | 1.28 (0.92, 1.88)^c^ | 1.18 (0.85, 1.72)^d^ | <0.001 |
| HDL_C mmol/L | 1.52 ± 0.43^a^ | 1.52 ± 0.42^a^ | 1.51 ± 0.42^a^ | 1.52 ± 0.41^a^ | <0.001 |
| LDL_C mmol/L | 2.63 ± 0.79^a,b^ | 2.61 ± 0.79^a,c^ | 2.61 ± 0.81^b,c^ | 2.56 ± 0.82 | <0.001 |
| UA μmmol/L | 297.3 ± 88.9^a^ | 289.9 ± 86.6^b^ | 287.6 ± 86.3^c^ | 281.4 ± 83.4^d^ | <0.001 |
| FBG mmol/L | 5.70 ± 1.50^a^ | 5.68 ± 1.55^a^ | 5.63 ± 1.45^b^ | 5.60 ± 1.42^b^ | <0.001 |
| SBP mmHg | 131.3 ± 18.8^a^ | 131.6 ± 19.0^a^ | 131.4 ± 19.5^a^ | 131.2 ± 20.1^a^ | <0.001 |
| BRI_06_ | 3.89 ± 0.95^a^ | 3.79 ± 1.01^b^ | 3.68 ± 1.09^c^ | 3.70 ± 1.66^c^ | <0.001 |
| BRI (CV) | 6.18 (4.27, 7.81)^a^ | 12.3 (10.8, 13.7)^b^ | 18.8 (16.9, 20.8)^c^ | 30.2 (26.2, 37.0)^d^ | <0.001 |
| BRI (SD) | 0.23 (0.15, 0.30)^a^ | 0.46 (0.38, 0.55)^b^ | 0.70 (0.58, 0.83)^c^ | 1.13 (0.91,1.45)^d^ | <0.001 |
| BRI (VIM)  Time-averaged BRI  Cumulative BRI  Diabetes, N (%) | 0.23 (0.16, 0.30)^a^  3.95 ± 0.94  5762.1 ± 1539.0  1,384 (11.7) | 0.47 (0.41, 0.54)^b^  3.89 ± 0.96  5692 ±1546.1  1,355 (11.5) | 0.74 (0.65, 0.84)^c^  3.81 ± 0.95  5579.9 ± 1528.6  1,235 (10.5) | 1.22 (1.03, 1.52)^d^  1,176 (9.96) | <0.001  <0.001  <0.001  <0.001 |
| Hypertension, N (%) | 5,755 (48.7)^a^ | 5,629 (47.7)^a^ | 5,570 (46.3)^b^ | 5,180 (43.9)^c^ | <0.001 |
| Diabetes,N (%) | 1,384 (11.72)^a^ | 1,255 (11.47)^a^ | 1,235 (10.46)^b^ | 1,176 (9.96)^b^ | <0.001 |
| Antihypertensive treatment, N (%) | 2,027 (17.2)^a^ | 1,868 (15.8)^b^ | 1,642 (13.9)^c^ | 1,415 (12.0)^d^ | <0.001 |
| Lipid-lowering treatment, N (%) | 147 (1.24)^a,b^ | 143 (1.21)^a,c^ | 119 (1.01)^b,c^ | 88 (0.75) | <0.001 |
| Antidiabetic treatment, N (%) | 652 (5.52)^a^ | 629 (5.33)^a^ | 541 (4.58)^b^ | 505 (4.28)^b^ | <0.001 |
| Insulin, N (%) | 214 (1.81) | 225 (1.91)^a^ | 185 (1.57) | 168 (1.42)^b^ | 0.014 |
| Metformin, N (%) | 119 (1.01)^a,b^ | 114 (0.97)^a,c^ | 95 (0.80)^b,c^ | 77 (0.65) | 0.012 |
| Glinides, N (%) | 129 (1.09)^a^ | 137 (1.16)^a^ | 106 (0.90)^a^ | 100 (0.85)^a^ | 0.043 |
| PCM hypoglycemic agents, N (%) | 39 (0.33)^a^ | 59 (0.50)^a^ | 53 (0.45)^a^ | 49 (0.41)^a^ | 0.235 |
| Others, N (%) | 234 (35.89)^a,b^ | 241 (38.31)^a,c^ | 199 (36.78)^b,c^ | 185 (36.63) | 0.839 |

Data are presented as mean ± standardized deviation, median (p25, p75), or n(percentage). SBP, systolic blood pressure; BMI, body mass index; FBG, fasting

blood glucose; LDL-C, low-density lipoprotein cholesterol; HDL-C, high-density lipoprotein cholesterol; TG, triglyceride; UA, uric acid; BRI, Body roundness

index; BRI_06_, Body roundness index in 2006-2007; ARV, average real variability; VIM, variation independent of mean; CV, coefficient of variation; SD,

standard deviation; PCM, Proprietary Chinese medicine; Superscript letters (a, b, c, d) denote statistically significant pairwise differences between groups; groups

not sharing the same letter differ significantly (False Discovery Rate–adjusted *p* < 0.05).

**Supplement Table 3**. Joint associations of cumBRI and BRI variability with the incidence of fragility fractures

| **Index** | **Case/Total** | **Incidence rate^a^** | **Model** |
| --- | --- | --- | --- |
| **Combination of cumBRI and variability** | | | |
| **Cut points: median of cumBRI and CV** |  |  |  |
| Low cumBRI and low CV | 144/11,292 | 1.09 | Reference |
| Low cumBRI and high CV | 174/12,328 | 1.22 | 1.08 (0.87-1.35) |
| High cumBRI and low CV | 197/12,328 | 1.43 | 1.19 (0.94-1.51) |
| High cumBRI and high CV | 210/11,292 | 1.69 | 1.36 (1.08-1.71) |
| **Cut points: median of cumBRI and VIM** |  |  |  |
| Low cumBRI and low VIM | 122/9,849 | 1.05 | Reference |
| Low cumBRI and high VIM | 196/13,771 | 1.23 | 1.12 (0.89-1.41) |
| High cumBRI and low VIM | 218/13,771 | 1.41 | 1.19 (0.94-1.53) |
| High cumBRI and high VIM | 189/9,849 | 1.74 | 1.43 (1.12-1.83) |
| **Cut points: median of cumBRI and ARV** | | | |
| Low cumBRI and low ARV | 173/13,791 | 1.07 | Reference |
| Low cumBRI and high ARV | 145/9,829 | 1.28 | 1.16 (0.93-1.44) |
| High cumBRI and low ARV | 153/9,829 | 1.40 | 1.21 (0.95-1.54) |
| High cumBRI and high ARV | 254/13,791 | 1.67 | 1.38 (1.11-1.73) |
| **Cut points: median of cumBRI and SD** | | | |
| Low cumBRI and low SD | 174/13,840 | 1.07 | Reference |
| Low cumBRI and high SD | 144/9,780 | 1.27 | 1.15 (0.92-1.44) |
| High cumBRI and low SD | 151/9,780 | 1.38 | 1.19 (0.94-1.52) |
| High cumBRI and high SD | 256/13,840 | 1.68 | 1.39 (1.12-1.74) |

BRI_06_: Body roundness index in 2006-2007; Incidence rate^a^ per 1000 person-years;Model: adjusted for age, sex, smoking status, drinking status,

intake salt status, education, physical activity, TG, HDL_C, LDL_C, UA, Antidiabetic agents, Antihypertensive agents, Lipid-lowering agents,

BMI in baseline, and BRI_06;_ median of cumBRI, 5470.86, median of variability CV, VIM, ARV, and SD are 15.268, 0.594, 0.668, and 0.561, respectively

**Supplement Table 4** . Reclassification and discrimination statistics

| **Models** | C-index (95% CI) | Integrated Discrimination Improvement (95% CI) | *P* value | Net Reclassification Index (95% CI) | *P* value |
| --- | --- | --- | --- | --- | --- |
| Model | 0.604 (0.5823-0.6251) | Reference |  | Reference |  |
| Model+ BRI | 0.608 (0.5870-0.6290) | 0.000086 (0-0.0002) | 0.077 | 0.071 (-0.002-0.145) | 0.057 |
| Model+ Height | 0.604 (0.5825-0.6252) | -0.000007 (0-0) | 0.011 | -0.033 (-0.106-0.041) | 0.385 |
| Model+ Weight | 0.605 (0.5838-0.6264) | -0.000009 (0-0) | 0.460 | 0.013 (-0.060-0.086) | 0.349 |
| Model+ Waist circumference | 0.608 (0.5876-0.6299) | 0.00006 (0-0.0002) | 0.208 | 0.049 (-0.025-0.122) | 0.193 |
| Model+ Body mass index | 0.608 (0.5876-0.6299) | 0.00006 (0-0.0002) | 0.208 | 0.049 (-0.025-0.122) | 0.193 |
| Model+ TWA BRI | 0.609 (0.5882-0.6305) | 0.00012 (0-0.0002) | 0.028 | 0.091 (0.018-0.165) | 0.015 |
| Model+ BRI variability | 0.607 (0.5854-0.6278) | 0.00012(0-0.0002) | 0.013 | 0.100 (0.027-0.173) | 0.008 |
| Model+ TWA BRI+ BRI variability | 0.611 (0.5897-0.6318) | 0.00020 (0.0001-0.0003) | 0.003 | 0.117 (0.043-0.190) | 0.002 |

Note: BRI, body roundness index; BRI, height, weight, waist circumference, and body mass index were single time point at baseline; BRI variability measured by standard deviation. Model adjusted for age, sex, smoking status, drinking status, intake salt status, education, physical activity, TG, HDL_C, LDL_C, UA, antidiabetic agents, antihypertensive agents, lipid-lowering agents.

**Supplement Table 5**. Sensitivity analyses for the association between TWA BRI and the risk of fragility fractures

| **Index** | **Model ^a^** | **Model ^b^** | **Model ^c^** | **Model ^d^** |
| --- | --- | --- | --- | --- |
| **Time-weighted average BRI** |  |  |  |  |
| Quartile 1 | Reference | Reference | Reference | Reference |
| Quartile 2 | 1.13 (0.90-1.43) | 1.2(0.96-1.53) | 1.15 (0.92-1.45) | 1.15 (0.91-1.45) |
| Quartile 3 | 1.32 (1.03-1.68) | 1.36 (1.06-1.74) | 1.34 (1.04-1.69) | 1.31(1.03-1.67) |
| Quartile 4 | 1.41 (1.06-1.86) | 1.52 (1.15-2.01) | 1.52 (1.15-1.99) | 1.49 (1.13-1.96) |
| **Cumulative burden of BRI** |  |  |  |  |
| ≤0 | Reference | Reference | Reference | Reference |
| >0 | 1.36 (1.06-1.75) | 1.34 (1.05-1.73) | 1.38 (1.08-1.75) | 1.36 (1.07-1.73) |
| **Time exposure duration** |  |  |  |  |
| 0 year | Reference | Reference | Reference | Reference |
| 2 years | 1.14 (0.93-1.41) | 1.23 (1.00-1.51) | 1.20 (0.98-1.47) | 1.19 (0.98-1.46) |
| ≥ 4 years | 1.51 (1.16-1.97) | 1.54 (1.18-2.00) | 1.55 (1.20-2.0) | 1.52 (1.18-1.97) |

Model: adjusted for age, sex, smoking status, drinking status, intake salt status, education, physical activity, TG, HDL_C, LDL_C, UA, Antidiabetic agents, Antihypertensive agents, Lipid-lowering agents and BMI in baseline.

a Sensitivity analysis was performed by excluding participants use of Antidiabetic agents.

b Sensitivity analysis was performed by excluding outcome within the first year of follow-up

c Sensitivity analysis was performed using competing risk model considering death as a competing risk.

d Sensitivity analysis was performed further adjusted for diabetes.

**Supplement Table 6**. Sensitivity analyses for the association between variability of BRI and the risk of fragility fractures

| **Index** | **Model ^a^** | **Model ^b^** | **Model ^c^** | **Model ^d^** |
| --- | --- | --- | --- | --- |
| **CV** |  |  |  |  |
| Quartile 1 | Reference | Reference | Reference | Reference |
| Quartile 2 | 1.17 (0.94-1.46) | 1.24 (1.00-1.54) | 1.18 (0.95-1.46) | 1.24 (0.99-1.54) |
| Quartile 3 | 1.08 (0.86-1.35) | 1.17 (0.93-1.46) | 1.13 (0.91-1.40) | 1.17 (0.93-1.46) |
| Quartile 4 | 1.27 (1.02-1.57) | 1.30 (1.04-1.62) | 1.29 (1.05-1.60) | 1.30 (1.04-1.62) |
| **VIM** |  |  |  |  |
| Quartile 1 | Reference | Reference | Reference | Reference |
| Quartile 2 | 1.09 (0.88-1.37) | 1.20 (0.97-1.50) | 1.12 (0.91-1.39) | 1.20 (0.97-1.50) |
| Quartile 3 | 1.24 (1.00-1.54) | 1.27 (1.02-1.58) | 1.25 (1.02-1.55) | 1.27 (1.02-1.58) |
| Quartile 4 | 1.16 (0.93-1.45) | 1.20 (0.96-1.51) | 1.18 (0.95-1.47) | 1.20 (0.96-1.51) |
| **ARV** |  |  |  |  |
| Quartile 1 | Reference | Reference | Reference | Reference |
| Quartile 2 | 1.16 (0.93-1.45) | 1.17 (0.93-1.46) | 1.18 (0.95-1.46) | 1.16 (0.93-1.46) |
| Quartile 3 | 1.22 (0.98-1.52) | 1.25 (1.00-1.56) | 1.13 (0.91-1.40) | 1.24 (0.99-1.55) |
| Quartile 4 | 1.26 (1.01-1.57) | 1.31 (1.05-1.64) | 1.29 (1.05-1.60) | 1.30 (1.04-1.62) |
| **SD** |  |  |  |  |
| Quartile 1 | Reference | Reference | Reference | Reference |
| Quartile 2 | 1.22 (0.98-1.53) | 1.26 (1.00-1.58) | 1.26 (1.02-1.57) | 1.25 (1.00-1.57) |
| Quartile 3 | 1.26 (1.01-1.57) | 1.28 (1.02-1.60) | 1.29 (1.04-1.61) | 1.28 (1.02-1.60) |
| Quartile 4 | 1.31 (1.05-1.64) | 1.36 (1.09-1.70) | 1.39 (1.12-1.72) | 1.35 (1.08-1.69) |

Model: adjusted for age, sex, smoking status, drinking status, intake salt status, education, physical activity, TG, HDL_C, LDL_C, UA, Antidiabetic agents, Antihypertensive agents, Lipid-lowering agents and BMI in baseline. a, Sensitivity analysis was performed by excluding participants use of Antidiabetic agents; b, Sensitivity analysis was performed by excluding outcome within the first year of follow-up; c, Sensitivity analysis was performed using competing risk model considering death as a competing risk. d, Sensitivity analysis was performed further adjusted for diabetes.

**Supplement Table 7**. Sensitivity analyses for joint associations between TWA BRI and BRI variability with fragility fracture incidence

| **Index** | **Model ^a^** | **Model ^b^** | **Model ^c^** | **Model ^d^** |
| --- | --- | --- | --- | --- |
| **Combination of Time-weighted average BRI and variability** | | |  |  |
| **Cut points: median of TWA BRI and VIM** |  |  |  |  |
| Low TWA BRI and low VIM | Reference | Reference | Reference | Reference |
| Low TWA BRI and high VIM | 1.23 (0.98-1.56) | 1.22 (0.96-1.55) | 1.23 (0.98-1.55) | 1.21 (0.96-1.54) |
| High TWA BRI and low VIM | 1.34 (1.04-1.73) | 1.36 (1.05-1.75) | 1.36 (1.06-1.75) | 1.34 (1.04-1.73) |
| High TWA BRI and high VIM | 1.49 (1.16-1.93) | 1.47 (1.14-1.91) | 1.53 (1.19-1.97) | 1.46 (1.13-1.89) |
| **Cut points: median of TWA BRI and CV** |  |  |  |  |
| Low TWA BRI and low CV | Reference | Reference | Reference | Reference |
| Low TWA BRI and high CV | 1.18 (0.94-1.47) | 1.18 (0.94-1.49) | 1.18 (0.95-1.48) | 1.18 (0.94-1.49) |
| High TWA BRI and low CV | 1.35 (1.06-1.73) | 1.34 (1.05-1.72) | 1.35 (1.06-1.72) | 1.33 (1.04-1.70) |
| High TWA BRI and high CV | 1.37 (1.08-1.75) | 1.40 (1.10-1.79) | 1.44 (1.143-1.82) | 1.39 (1.08-1.77) |
| **Cut points: median of TWA BRI and ARV** |  |  |  |  |
| Low TWA BRI and low ARV | Reference | Reference | Reference | Reference |
| Low TWA BRI and high ARV | 1.20 (0.96-1.50) | 1.23 (0.98-1.54) | 1.21 (0.97-1.51) | 1.23 (0.98-1.54) |
| High TWA BRI and low ARV | 1.31 (1.03-1.67) | 1.31 (1.03-1.69) | 1.32 (1.04-1.68) | 1.29 (1.01-1.66) |
| High TWA BRI and high ARV | 1.38 (1.10-1.74) | 1.43 (1.14-1.80) | 1.44 (1.15-1.80) | 1.42 (1.12-1.78) |
| **Cut points: median of TWA BRI and SD** |  |  |  |  |
| Low TWA BRI and low SD | Reference | Reference | Reference | Reference |
| Low TWA BRI and high SD | 1.25 (1.00-1.56) | 1.22 (0.98-1.54) | 1.25 (1.01-1.56) | 1.22 (0.97-1.53) |
| High TWA BRI and low SD | 1.35 (1.06-1.73) | 1.31 (1.02-1.68) | 1.35 (1.06-1.72) | 1.30 (1.01-1.67) |
| High TWA BRI and high SD | 1.40 (1.11-1.7) | 1.42 (1.13-1.79) | 1.46 (1.17-1.83) | 1.41 (1.12-1.77) |

Model: adjusted for age, sex, smoking status, drinking status, intake salt status, education, physical activity, TG, HDL_C, LDL_C, UA, Antidiabetic agents, Antihypertensive agents, Lipid-lowering agents and BMI in baseline. a, Sensitivity analysis was performed by excluding participants use of Antidiabetic agents; b, Sensitivity analysis was performed by excluding outcome within the first year of follow-up; c, Sensitivity analysis was performed using competing risk model considering death as a competing risk. d, Sensitivity analysis was performed further adjusted for diabetes.


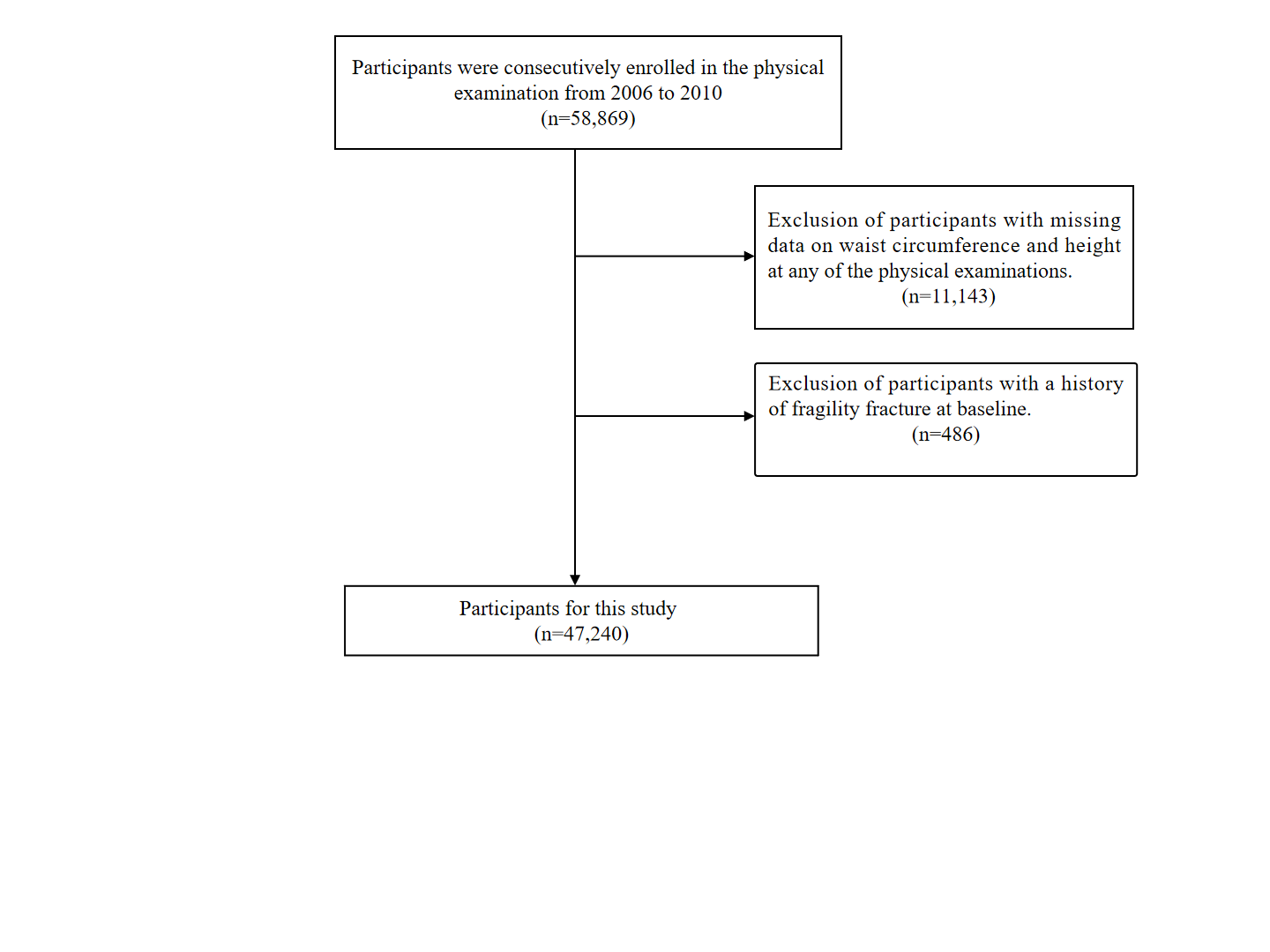


**Supplement Figure 1**. Flow chart of study participants.


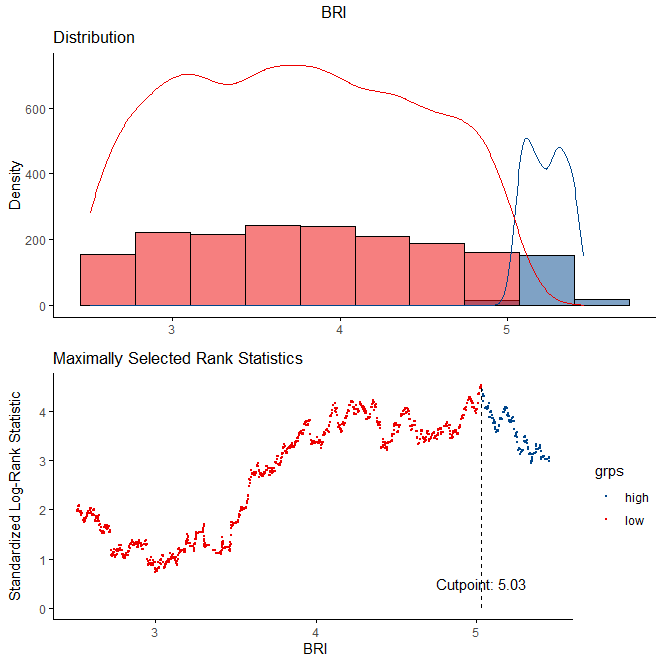


**Supplement Figure 2**. Distribution of BRI and determination of the optimal cutpoint using maximally selected rank statistics.


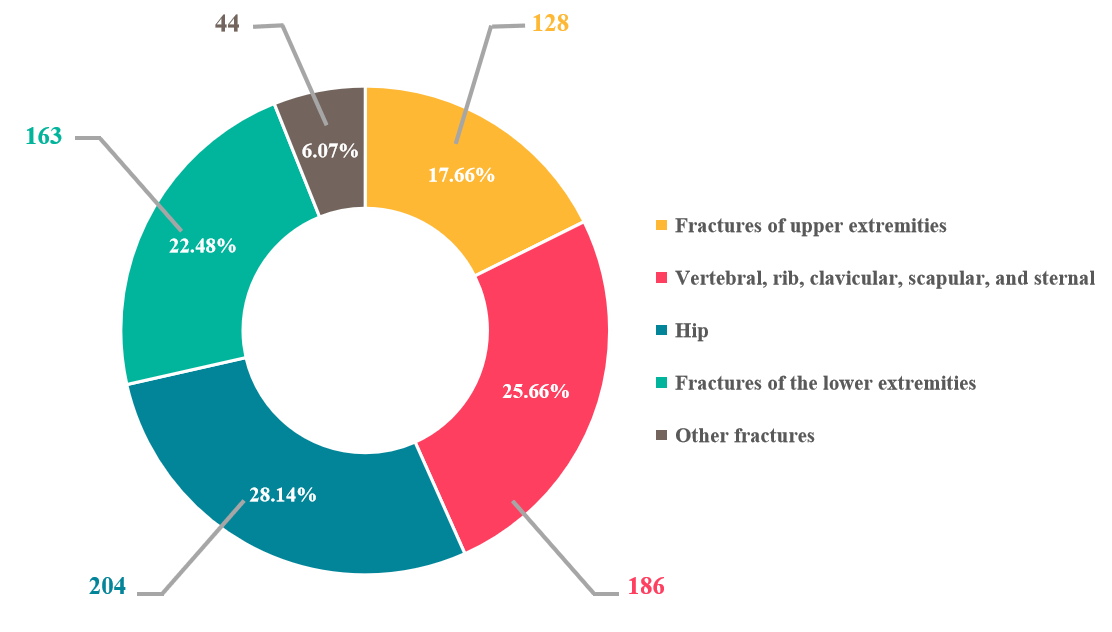


**Supplement Figure 3**. Distribution of fractures location


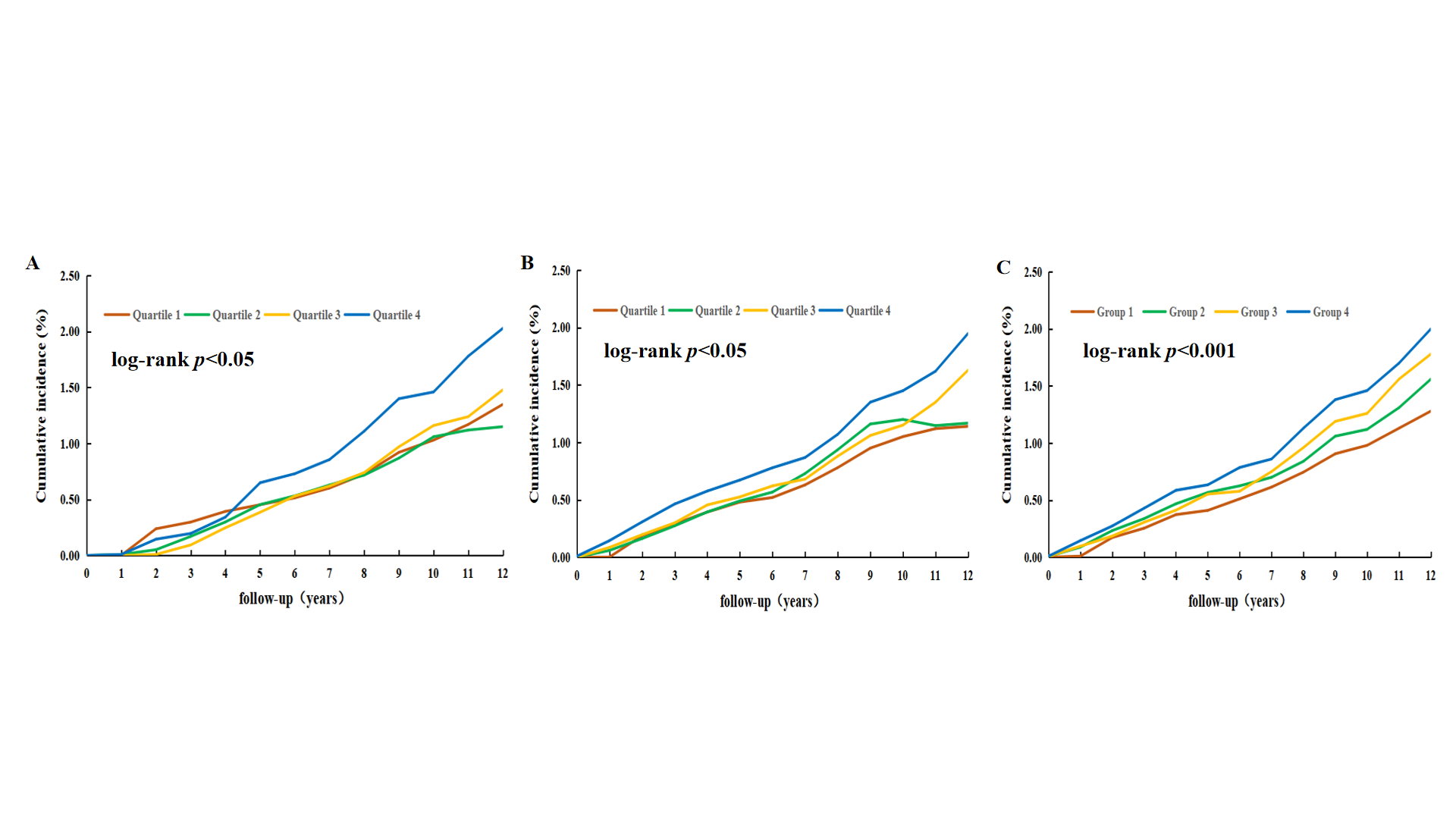


**Supplement Figure 4**. Cumulative incidence of fragility fractures

**a**, Cumulative incidence of fragility fracture in different TWA BRI groups;

**b**, Cumulative incidence of fragility fracture in in BRI different variability (measured by CV) groups

**c**, Cumulative incidence of fragility fracture in different joint associations of TWA BRI and BRI variability groups.

**Supplement Figure 5**. Stratified association between cumulative BRI indexes with fragility Fracture Incidence by age and sex

Model adjusted for age, sex, smoking status, drinking status, intake salt status, education, physical activity, TG, HDL_C, LDL_C, UA, antidiabetic agents, antihypertensive agents, lipid-lowering agents, and BMI in baseline.


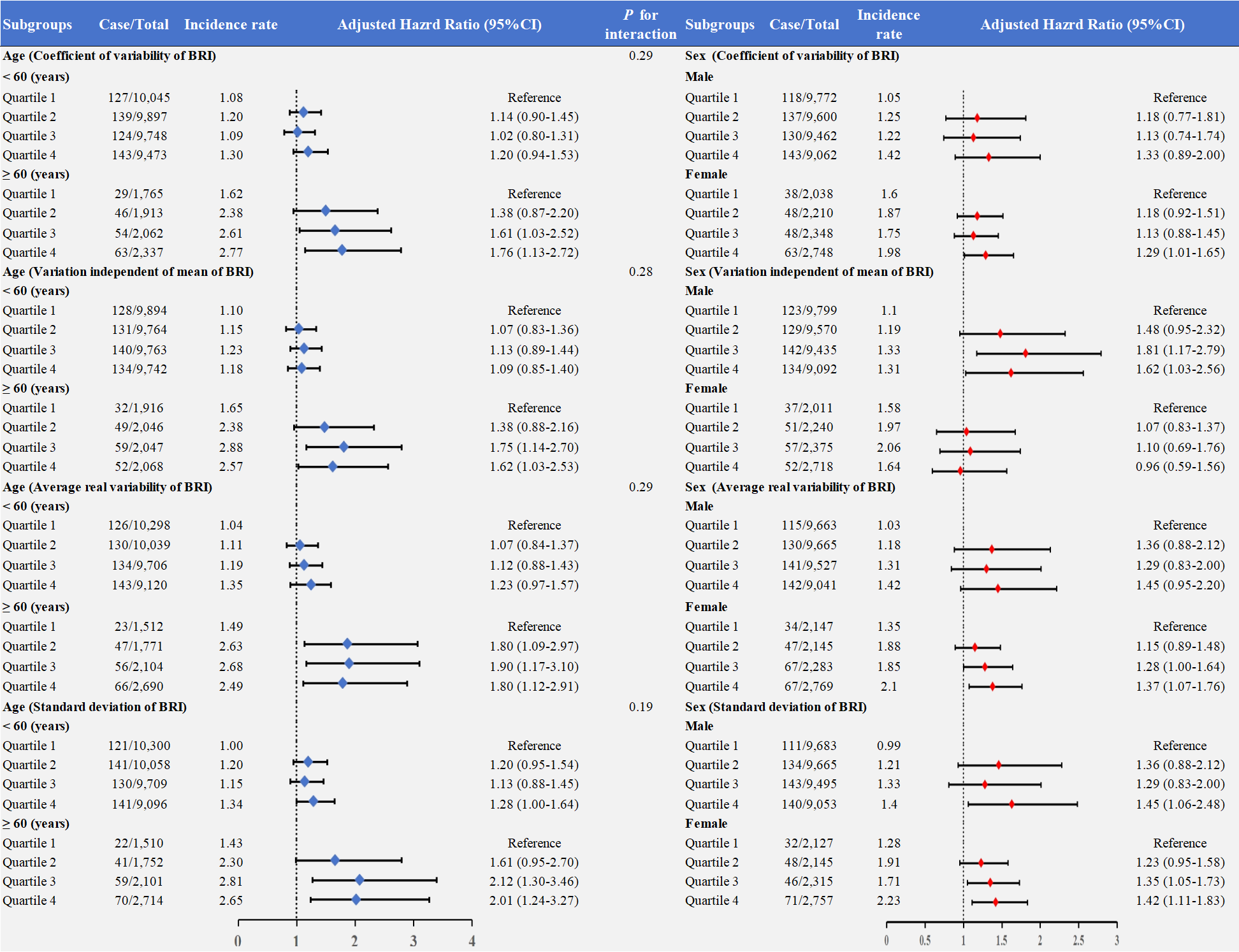


**Supplement Figure 6**.Stratified association between BRI variability indexes with fragility Fracture Incidence by age and sex

Model adjusted for age, sex, smoking status, drinking status, intake salt status, education, physical activity, TG, HDL_C, LDL_C, UA,

antidiabetic agents, antihypertensive agents, lipid-lowering agents, and BMI in baseline.


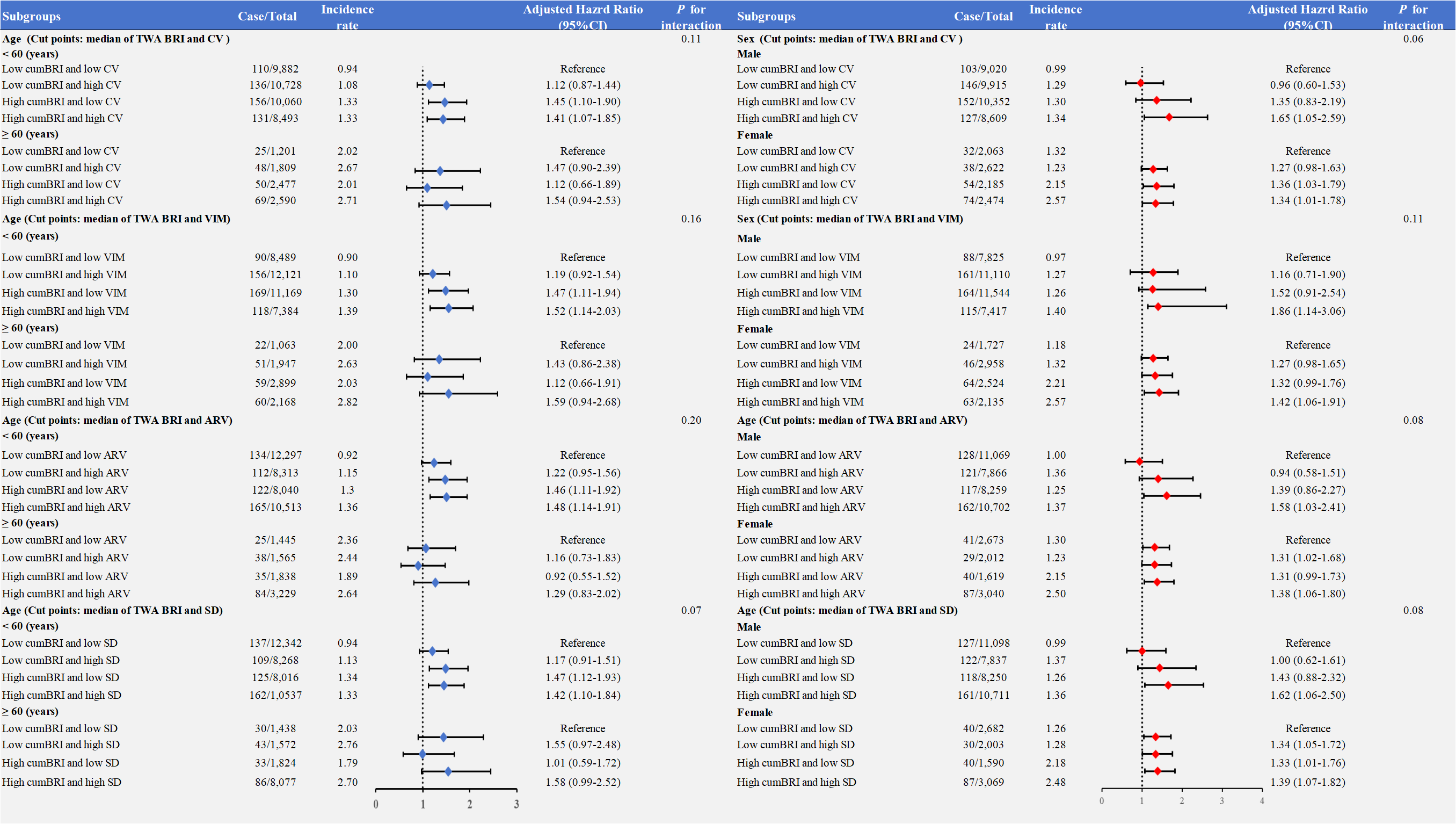


**Supplement Figure 7**. Stratified analyses for joint associations between TWA BRI and BRI variability with fragility fracture

incidence by age and sex.

Model adjusted for age, sex, smoking status, drinking status, intake salt status, education, physical activity, TG, HDL_C, LDL_C, UA,

antidiabetic agents, antihypertensive agents, lipid-lowering agents, and BMI in baseline.
